# QSPR Models for Predicting Log P_liver_ Values for Volatile Organic Compounds Combining Statistical Methods and Domain Knowledge

**DOI:** 10.3390/molecules171214937

**Published:** 2012-12-17

**Authors:** Damián Palomba, María J. Martínez, Ignacio Ponzoni, Mónica F. Díaz, Gustavo E. Vazquez, Axel J. Soto

**Affiliations:** 1 Planta Piloto de Ingeniería Química (PLAPIQUI) CONICET-UNS, La Carrindanga km.7, Bahía Blanca, 8000, Argentina; E-Mails: dpalomba@plapiqui.edu.ar (D.P.); ip@cs.uns.edu.ar (I.P.); mdiaz@plapiqui.edu.ar (M.F.D.); 2 Laboratorio de Investigación y Desarrollo en Computación Científica (LIDeCC), DCIC, UNS, Av. Alem 1250, Bahía Blanca, 8000, Argentina; E-Mails: mjma@cs.uns.edu.ar (M.J.M.); gev@cs.uns.edu.ar (G.E.V.); 3 Faculty of Computer Science, Dalhousie University, 6050 University Av., PO BOX 15000, Halifax, NS B3H 4R2, Canada

**Keywords:** log P_liver_, VOCs, machine learning, QSPR

## Abstract

Volatile organic compounds (VOCs) are contained in a variety of chemicals that can be found in household products and may have undesirable effects on health. Thereby, it is important to model blood-to-liver partition coefficients (log P_liver_) for VOCs in a fast and inexpensive way. In this paper, we present two new quantitative structure-property relationship (QSPR) models for the prediction of log P_liver_, where we also propose a hybrid approach for the selection of the descriptors. This hybrid methodology combines a machine learning method with a manual selection based on expert knowledge. This allows obtaining a set of descriptors that is interpretable in physicochemical terms. Our regression models were trained using decision trees and neural networks and validated using an external test set. Results show high prediction accuracy compared to previous log P_liver_ models, and the descriptor selection approach provides a means to get a small set of descriptors that is in agreement with theoretical understanding of the target property.

## 1. Introduction

Volatile organic compounds (VOCs) are emitted as gases from certain solids or liquids. VOCs include a variety of chemicals, some of which may have short- and long-term adverse health effects. Concentrations of many VOCs are consistently higher indoors (up to ten times higher) than outdoors. Organic chemicals are widely used as ingredients in household products. Paints, varnishes, and wax all contain organic solvents, as do many cleaning, disinfecting, cosmetic, degreasing, and hobby products. All of these products may release organic compounds while they are used, and, to some degree, when they are stored. The main concern is the potential for VOCs to adversely impact on the health of people that are exposed to them indoors [1,2]. Woodruff *et al.* [3] described the need for better public health policies on chemicals released into our environment. They proposed modernizing approaches to assessing health risk and remarked the importance of scientific understanding of the relationship between pollutant exposure and adverse health effects.

In this context, quantitative structure-property relationship (QSPR) models allow one to relate measurements on a set of “descriptor” (or predictor) variables to the behavior of the response variable and constitute a valuable tool for *in silico* property prediction. In particular, the development of combinatorial chemistry and high throughput screening programs has stimulated drug discovery research to find theoretical and computational models to estimate and predict drug absorption, distribution, metabolism, and excretion (ADME) based on drug physicochemical properties [4]. These methodologies have also been applied to VOCs inhalation studies [5,6] and are related to the analysis of physiologically-based pharmacokinetic (PBPK) models. 

PBPK modeling is a mathematical modeling technique for predicting the ADME of synthetic or natural chemical substances in humans and other animal species. In respiratory PBPK models blood-air, liver-air and liver-blood partition coefficients of VOCs are important for their hazard assessment and bioavailability estimation [7]. Several attempts have been made to model the relationship between the structure or molecular properties and the blood-to-liver distribution, usually denoted as log P_liver_, of VOCs and drugs. Abraham and Weathersby [8] used the Abraham descriptors to estimate values of log P_liver_ of VOCs. Balaz and Luckacova [9] correlated values of log P_liver_ for 28 compounds by using four variables. Poulin and Theil [10,11] developed an equation for the prediction of *in vivo* plasma-to-tissue partition coefficients of drugs. Zhang [12] built a nonlinear model to calculate log P_liver_ of VOCs. Liu *et al.* [13] obtained a nonlinear model for predicting the tissue-to-blood partition of organic compounds using a least squares support vector machine. Rodgers *et al.* [14] achieved equations for the prediction of plasma-water-to-tissue distribution. Zhang and Zhang [15] generated a general training model for predicting *in vivo* blood-to-liver (among other tissues) distribution of drugs. Abraham *et al.* [7] applied solvation equations to correlate *in vitro* blood-to-liver partition coefficients for VOCs and drugs. Martín-Biosca *et al.* [16] employed biopartitioning micellar chromatography (BMC) for predicting blood-to-tissue partition coefficients of drugs and proposed PLS2 and multiple linear regression (MLR) models based on BMC retention data.

While most of these works make interesting contributions to the study of the log P_liver_ property, in general their predictive accuracies or chemical interpretation are not good enough for wide use at an industrial scale. In particular, a key issue for data-driven QSPR methodologies is how expert knowledge can be incorporated into the modeling process in order to obtain interpretable predictors. For these reasons, new statistical QSPR models for log P_liver_ addressing these premises are presented in this work. The proposed methodology combines the use of machine learning methods with expert analysis for the identification of the most relevant molecular descriptors for the definition of the QSPR model. This integration is achieved by means of a careful analysis, where a reduced number of descriptors selected by data-driven methods are evaluated by experts in terms of their chemical meaning and statistical contribution to a candidate QSPR model. From this semi-automatic analysis a new set of descriptors is chosen, and hence the associated statistical QSPR model is finally obtained. In this way, a double contribution is pursued in this work. First, the design of new log P_liver_ models with high prediction accuracy and good interpretability. Second, the application of our specific design methodology that integrates machine learning with human expert knowledge, and hence recommending its analogous applications for prediction of other chemical properties.

The article is structured as follows: in Section 2, the main results obtained from a log P_liver_ dataset are presented. Section 3 describes the methodological approach applied for our experiments, and it also includes a thorough analysis of the contribution of the descriptors used in our models. Finally, in Section 4 main conclusions of this work are discussed.

## 2. Results and Discussion

### 2.1. Dataset and Calculation of the Molecular Descriptors

The *in vitro* blood-to-liver partition coefficients, log P_liver_ (human/rat), values were taken from Abraham *et al.* [7]. In this data set there are 122 VOCs among which are hydrocarbons, alkyl halides, alcohols, ethers, esters, ketones, epoxides, nitriles, halobenzenes, polycyclic hydrocarbons and benzene derivatives (Table 1). The values of log P_liver_ range from −0.56 to 1.17.

A critical step in the development of QSPR models is the computation of the molecular descriptors. The model performance and results are strongly dependent on the way descriptors are calculated. The calculation process of the molecular descriptors is described as follows: all VOCs structures were drawn using HyperChem 8.0.7 [17]. The molecules were optimized with the same software, in order to find energetically stable conformations. The structures were pre-optimized with the Force Field Molecular Mechanics (MM+) procedure. Then, the resulting geometries were further refined by means of the Semi-Empirical Molecular Orbital Method AM 1 (Austin Model 1) by using Polak-Ribiere’s algorithm and a gradient norm limit of 0.01 kcal/(Å mol). As a next step, the HyperChem output files were used by Dragon 5.5 [18,19] to calculate several classes of descriptors such as: constitutional, geometrical, topological and electrostatic. Finally, constant descriptors (*i.e.*, variables that take a same value for all samples in the dataset) and near constants (*i.e.*, variables that take a same value, but allowing some predetermined small number of samples to take other values) were deleted.

### 2.2. Performance of Our Model

In order to evaluate the prediction capacity of our methodology, two different experiments were carried out in this work. The first experiment reports the performance of our models when tested on one sixth of the dataset (16.6%). When using decision trees, the mean absolute error (MAE) is 0.15 ± 0.04 (“±values” correspond to the confidence intervals calculated at 95% level). The root mean squared error (RMSE) is 0.18 and the coefficient of determination (R^2^) is 0.73.

**Figure 1:**
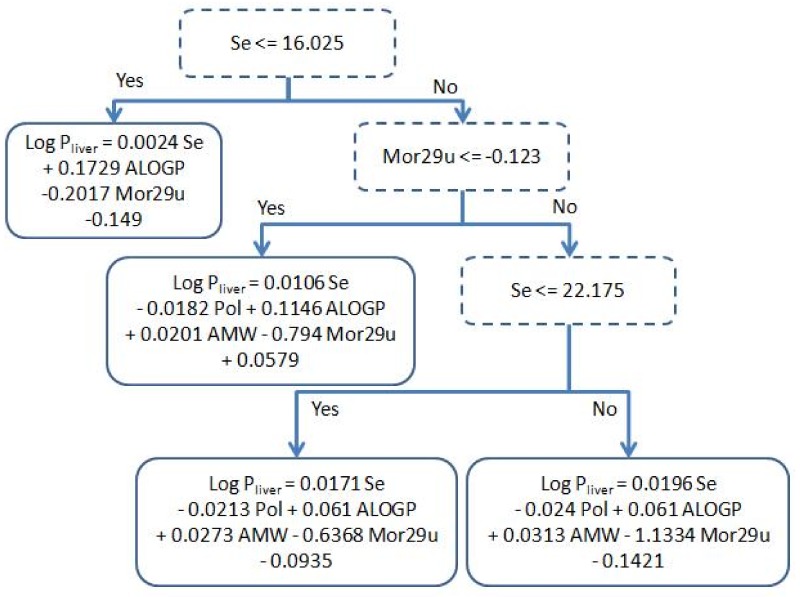
Decision tree model obtained after holding out 16.6% using M5*p* algorithm.

**Figure 2:**
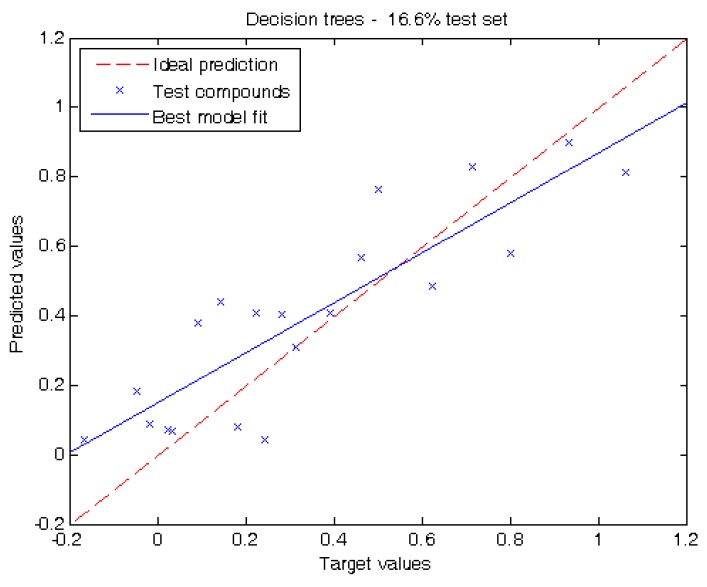
Target values *vs.* predicted values using 16.6% of the compounds for testing using the decision tree depicted in Figure 1.

Figure 1 shows the conditions on the internal nodes of the decision tree and the linear regressions used in the leaves, while Figure 2 shows a plot displaying the prediction of each individual test compound with the best linear fit of our model. The analysis of the tree structure sheds light on the understanding of the model used for prediction. The first decision of the tree is based on the value of Se; if it is lower than 16.025, this leads to a leaf with a simple regression using only three out of the five available descriptors, namely: ALOGP, Mor29u and Se. Making a structural inspection of the compounds that are associated to this leaf, it can be appreciated (Table S3, in supplementary material) that most of them have a short carbon chain and halogens with low log P_liver_ values. This separation is coherent with a physicochemical point of view: small polar molecules have higher affinity with blood mediums than longer ones. Another observation is that AMW and Pol have a rather high Pearson correlation (|r| ≈ 0.56) to Se (Table 2) and hence their contributions can be mainly explained by Se.

When the value of Se is greater than 16.025, there are three different linear regressions using the five descriptors. From Table 2, we can see that the correlation of AMW and Pol to Se are much lower than what happens in the left branch, and hence they now become necessary in the model. Note that all coefficients retain the same sign, indicating that the contribution of the descriptors to the model is always the same, and the differences in the coefficients come from producing a better fit to the compounds assigned to a specific leaf. We can also compare from Table 2, that ALOGP becomes more correlated to Se in the right branch than in the left one. Thereby, we can see in Figure 1 that there is a drop in the absolute value of the coefficient assigned to the ALOGP descriptor (0.061 and 0.1146) in the right branch compared to the one in the left branch (0.1729). A more thorough analysis of the physicochemical relevance of the descriptors can be found in Section 3.2.

Neural network ensemble on this same data partition reported a slight decrease of the regression accuracy compared to our previous model: MAE = 0.17 ± 0.04, RMSE = 0.19 and R^2^ = 0.66. The prediction obtained per compound in this experiment using decision trees and neural network ensemble can be found in Table 1.

For the training set, we obtained the following metrics using decision trees: MAE = 0.13 ± 0.02, RMSE = 0.17 and R^2^ = 0.75. Neural network ensemble reported MAE = 0.12 ± 0.02, RMSE = 0.16 and R^2^ = 0.80.

**Figure 3:**
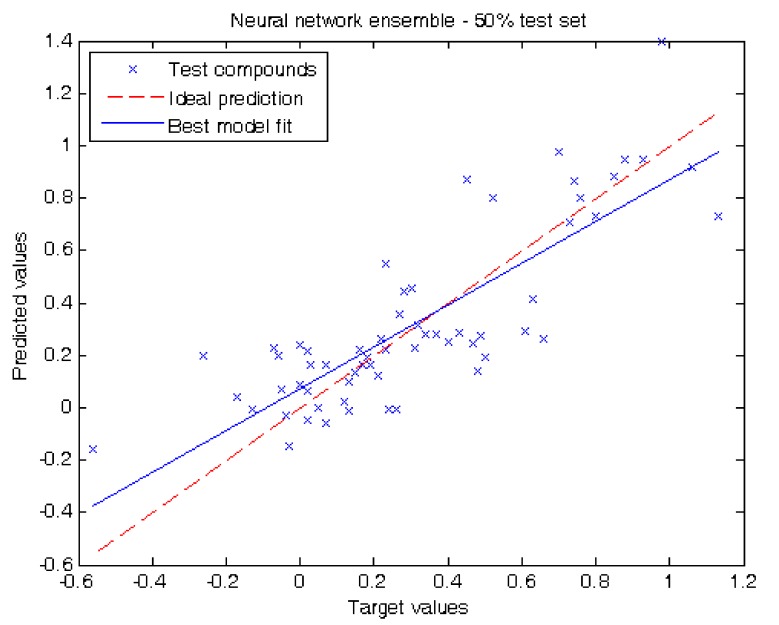
Target values *vs.* predicted values using 50.0% of the compounds for testing using neural network ensemble.

In our second experiment we evaluated our results by separating half of the compounds of the dataset for testing. When using decision trees we obtained the following metrics: MAE = 0.15 ± 0.04, RMSE = 0.21 and R^2^ = 0.62. Using neural network ensemble results in a higher prediction performance reporting MAE = 0.16 ± 0.03, RMSE = 0.20 and R^2^ = 0.66. Figure 3 shows the prediction values for each test compound using neural network ensemble. The prediction obtained per compound in this experiment using decision trees and neural network ensemble can be also found in Table 1.

When using decision trees we obtained MAE = 0.17 ± 0.03, RMSE = 0.21 and R^2^ = 0.62 for the training set. Neural network ensemble on this same partition reported MAE = 0.11 ± 0.03, RMSE = 0.15 and R^2^ = 0.81. These last results show an improvement over the results published by Abraham *et al*. [7] as their experiments using the same dataset and the same test set size yielded an RMSE = 0.221 and R^2^ = 0.481.

## 3. Computational Methods and Experiments 

In order to select the most relevant descriptors, a mixed scheme of automatic and expert chemical knowledge was employed. As a first step a machine learning approach based on a cross-fold validation with in-fold feature selection was applied [20]. This approach consists in splitting the samples set into *n* folds. The feature selection uses a learning algorithm that is applied to predict each fold by using the samples in the *n-*1 remaining folds. Since *n* different sets of features can be selected a voting scheme is employed, where the most frequently selected descriptors are kept for the final set of relevant descriptors. This technique ensures that particular predictions are not biased by feature over-selection or over-fitting since each prediction is performed without using the test samples neither during the feature selection nor during the classifier building process. From these experiments, the most frequently selected descriptors were kept for the initial set of relevant descriptors.

**Figure 4:**
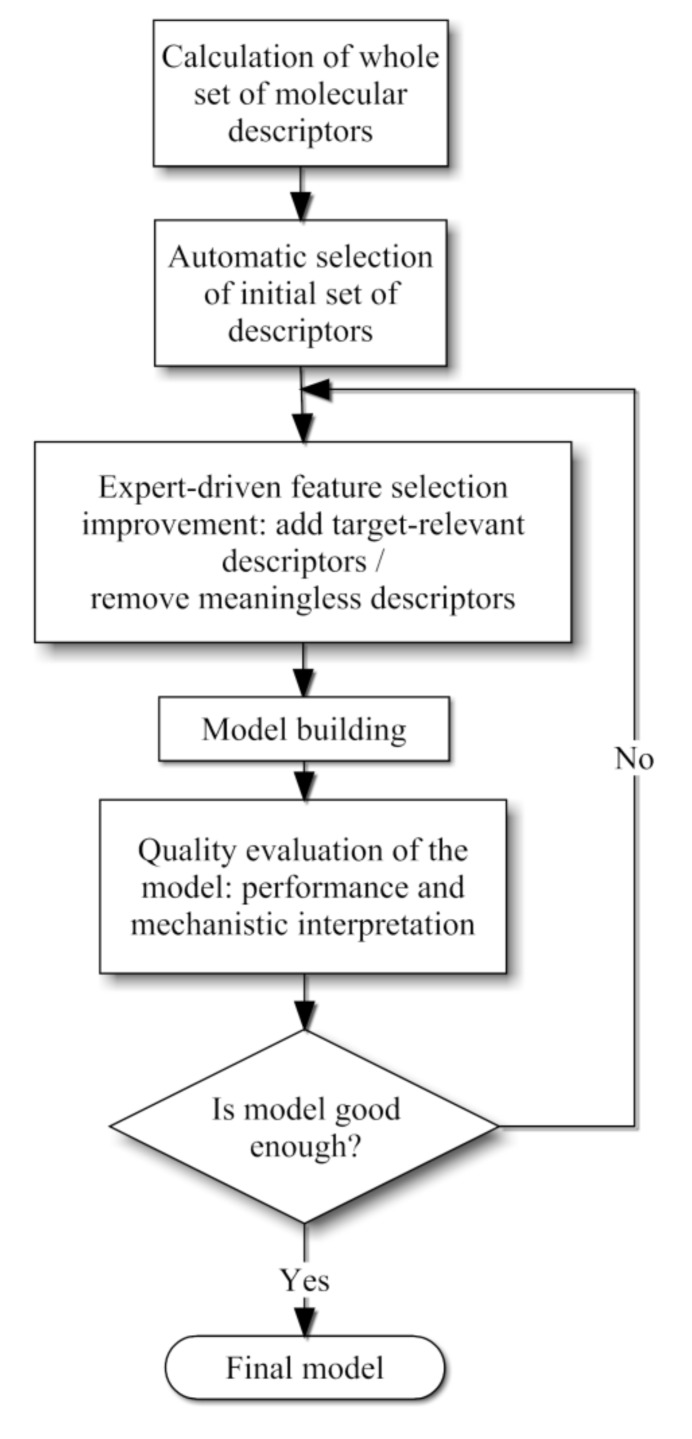
Combined methodology scheme proposed for the QSPR model development.

As a second step chemical knowledge was employed in order to evaluate the merit of each descriptor selected automatically. Since most of them did not exhibit a clear physicochemical explanation a small number of these descriptors were chosen for the final QSPR models, whereas other few descriptors were incorporated based on chemical expertise. Our methodology is schematized in Figure 4 and detailed explanations of these steps are given in the following subsections.

### 3.1. Molecular Descriptor Selection

The compounds listed in Section 2.1 were used to calculate 634 molecular descriptors using Dragon [18,19]. The final set of descriptors was chosen by using a combination of a feature selection method and a physicochemical-motivated strategy. The feature selection method that we used here is based on a 5-fold cross-validation with in-fold feature selection over the training set, which selected the following descriptors: RTu+, Mor29u, AMW, ZM2V, Jhetv, PW4, Ss, Ms, Me, Mv, nCIC, AAC, GATS2m, S1K, PW3, EEig07x, IC1, Qindex, RBN, Mor04m, Mor11v, ATS1v and MAXDN (complete names of the descriptors may be found in the E-Dragon web site [19]). After that, the physicochemical-motivated selection was done manually by domain experts, who aimed at including into the model orthogonal aspects of the molecules, so that important and interpretable features are considered and redundancy is kept minimal. These manually-selected descriptors are: AMW, Mor29u, ALOGP, Pol and Se; a brief description of each one is included in Table 3. The first two descriptors were taken from the feature selection algorithm results, and the following three were added on the basis of the experts’ criteria. Physicochemical rationale of this selection is supported in Section 3.2. Although this reduced subset of descriptors decreases the regression accuracy from R^2^ = 0.79, MAE = 0.13 ± 0.04 and RMSE = 0.15 to R^2^ = 0.73, MAE = 0.15 ± 0.04 and RMSE = 0.18 in our first experiment when a decision tree model is used, this subset is preferred for its low cardinality and more interpretable set of features. The values of the final pool of descriptors are available in the supplementary file (Table S3).

From the very beginning of our training process we held-out a test set of compounds, which is only used once to estimate an unbiased performance of our prediction method. We applied this validation strategy with two different sets of experiments. In the first experiment, we kept aside one sixth of the dataset (20 compounds) as a test set, whereas in the second experiment we used for testing half of the number of compounds in the dataset (61 compounds). In both cases the compounds selected for testing were chosen by using a stratified selection to ensure that compounds in the training and testing sets are similarly distributed.

Different machine learning methods such as linear regression, decision trees, neural network ensemble, SVM (support vector machine) and K-nearest neighbours were applied in this work, out of which decision trees and neural network ensemble stood out with the highest prediction accuracies for our dataset. All our experiments were run using data mining toolbox Weka [21]. In particular, the results with M5*p* (or M5*prime*) algorithm [22] and neural networks were discussed in this paper (Section 2). Details about the characteristics of these methods and their parameterization are explained in Section 3.3.

### 3.2. Physicochemical Relevance of Molecular Descriptors

The aim of this subsection is to analyze the relationship among molecular descriptors and the target property in order to provide a physicochemical justification of the resulting model. When the interpretation of a QSPR model is consistent with existing theories and knowledge of mechanisms, the model becomes more appealing for cheminformaticians [23]. Despite it is not always possible to find a global interpretation, it is desirable to make the effort to find an explanation for the model in a “mechanistic” way [24].

**Figure 5:**
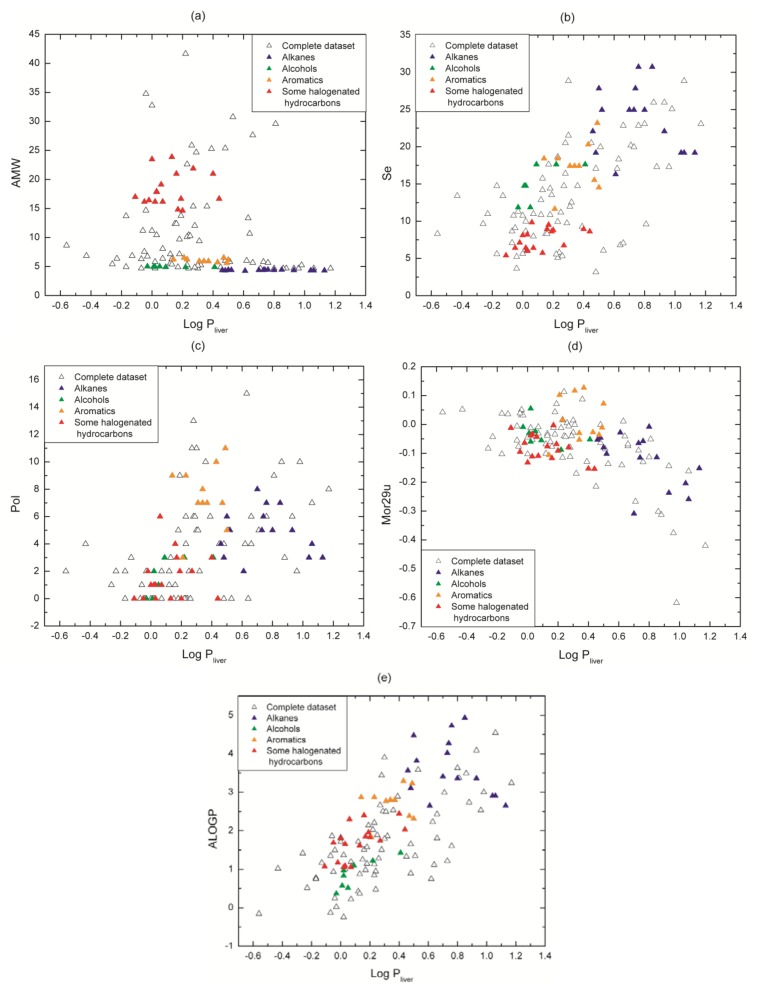
Plots of descriptors values *vs.* log P_liver_ values for the complete dataset. Some chemical families have been highlighted according to the color-coding presented in Table 1. (**a**) AMW; (**b**) Se; (**c**) Pol; (**d**) Mor29u and (**e**) ALOGP.

In our dataset values of log P_liver_ are consistent with regard to affinity for medium polarity, e.g., families with non-polar characteristic as alkanes (2 to 16 in Table 1) show higher affinity for liver tissue than for blood. The five descriptors chosen for the model provide to a lesser or greater extent important information about molecular properties related to the molecule capability to distribute between the two media under study: liver tissue and blood. The relationships between descriptor values and log P_liver_ values are shown in Figure 5. Our analysis is focused on some representative chemical families thathave been highlighted in colors in order to illustrate our point graphically (alkanes, alcohols, aromatics and some structurally similar halogenated hydrocarbons).

The descriptor AMW (molecular weight divided by the number of atoms) (Figure 5a) discriminates the molecules taking into account their atomic composition (type and quantity). Take for example the alkanes (C_n_ H_2n+2_) and the aromatics (C_n_ H_n_): they are constituted by carbons and hydrogens, and since each family has a different C/H rate, they present a specific value of AMW, even though the compounds are slightly different. When these families can be segregated from whole data set in the graph, the differences in their physicochemical properties become more evident, e.g., their polarity (which is related to the molecule affinity with an aqueous medium or a non-polar one). In this figure, it can also be seen the behavior of non-polar families as alkanes, where they tend to have high log P_liver_, while polar families as alcohols present lower log P_liver_. The same analysis can be applied to aromatics and halogenated hydrocarbons. Something similar happens to the descriptor Se (Figure 5b) that succeeds in discriminating the VOCs families with the sum of Sanderson atomic electronegativities (scaled on carbon atom).

The descriptors Pol (Polarity number) and Mor29u (3D- Molecule Representation of Structures Based on Electron diffraction - signal 29/unweighted) highlight structural 2D and 3D properties respectively and are plotted in Figure 5c,d. Pol relates to the steric properties of molecules and it is calculated on the distance matrix as the number of pairs of vertices at a topological distance equal to three (*i.e.*, number of third neighbors) [25]. In Figure 5c, it can be seen that Pol presents either low values or zero for short carbon chains whereas it takes higher values (between 4 and 16) for longer structures (e.g., most of the halogenated hydrocarbons and long alkanes respectively). In other words, Pol is low or equal to zero for compounds with few atoms because they have a small number of third neighbors and the opposite occurs for long molecules. Therefore, this descriptor works as a specific filter that discriminates molecules by chain length.

Mor29u (3D-MoRSE - signal 29/unweighted) belongs to 3D-MoRSE (3D-Molecule Representation of Structures based on Electron diffraction) descriptors. They are based on the idea of obtaining information from the 3D atomic coordinates by the transformation used in electron diffraction studies for preparing theoretical scattering curves [26]. 3D-MoRSE descriptors are derived from molecule atom projections along different angles, such as in electron diffraction. They represent different views of the whole molecule structure, although their meaning remains still unclear [27]. While its influence does not appear to be completely clear, its inclusion is based mainly upon a regression-based objective: it was selected by the feature selection method and in all our experiments, the removal of this descriptor from our equations lead to a remarkable drop in the train and testing prediction quality. Nevertheless, we can partially analyze its contribution. It can be seen in Figure 5d that Mor29u takes positive and negative values because the original equation includes the term *sin*(*s*·*r_ij_*)/*s*·*r_ij_* [26], where *s* measures the scattering angle and *r_ij_* represents the interatomic distances between atoms *i* and *j*. Then, the descriptor sign only is not determinant for the relationship with the target. Another observation from Figure 5d is that the chemical families are not segregated as occurs with AWM and Se (Figure 5a,b). This seems to be coherent because the components of a chemical family share many physicochemical properties (polarity, mobility, hydrogen bond, *etc*.) besides the 3D structure. Moreover, from Table S3 in supplementary material, it can be noted that isomers as o, m and p-xylenes, along with several examples, present different values, and thus they get differentiated. In brief, it is observed that Mor29u captures minimum variations in 3D-structural features based on interatomic distances.

Finally, ALOGP (Ghose-Crippen octanol-water partition coefficient) gives relevant information about molecular affinity for an octanol-water medium. In fact, ALOGP is a descriptor that commonly appears in models about partition coefficients [9,15]. It is calculated from a model consisting of a regression equation based on the hydrophobicity contribution of 120 atom types [28,29,30]. Each atom in every structure is classified into one of the 120 atom types. Then, an estimated log P value for any compound is given by ALOGP = $\underset{i}{\sum }n_{i}a_{i}$, where *n_i_* is the number of atoms of type *i* and *a_i_* is the corresponding hydrophobicity constant. 

It can be seen in Figure 5e that each VOC has its own ALOGP value regardless of its chemical family. That is, this descriptor is sensitive to minimum differences in molecular structure. As expected, it can be noted a correlation between this descriptor and log P_liver_ (Figure 5e), because polar molecules have low ALOGP and log P_liver_ values (e.g., alcohols and halogenated hydrocarbons) and non-polar ones have high values (e.g., alkanes and aromatics). 

### 3.3. Regression Algorithms

Two methodologies applied as regression algorithms, namely M5*p* and an ensemble of neural networks, were applied in this work. The decision tree model applied here is M5*p* [22]. This is an extension of Quinlan's M5 algorithm that allows using decision trees for regression problems, *i.e.*, attributes and target variable can be continuously defined over the set of real numbers. A key aspect of this decision tree algorithm is that it makes use of a linear regression model for each leaf of the tree. It also provides a mechanism for pruning (*i.e.*, keeping the height of the tree minimal to avoid overfitting) and a smoothing process that allows compensating discontinuities between adjacent linear models at the leaves of the tree. For our experiments we set to 4 the minimum allowed number of compounds per leaf. The neural networks used in our experiments make use of the traditional backpropagation algorithm, which was used before in the QSPR literature [31]. A total of fifty networks were used to define the ensemble. The architecture of each network is a single hidden layer with three nodes and all activation functions of the internal nodes of the network are sigmoids. The networks were initialized with different random weights. To facilitate the gradient optimization of the parameters all descriptors were normalized before training. The learning rate and the momentum were set to 0.3 and 0.2 respectively.

Neural networks and decision trees constitute very different modeling techniques in nature. On the one hand, neural networks are one of the most popular techniques for QSPR modeling and are able to fit any kind of function, provided there is a sufficient number of hidden nodes. This aspect also makes them prone to overfit the training data very easily (in the absence of any mechanism to thwart overfitting). On the other hand, decision trees are well accepted by lay users, who are able to interpret the meaning of the model very easily. Therefore, a decision on which of these models should be used would be based on how important the understanding of the prediction model is.

## 4. Conclusions 

In this paper we introduced new models for the prediction of blood-to-liver partition coefficients for volatile organic compounds following a QSPR approach. We applied two different machine learning approaches to model log P_liver_, namely: decision trees and neural networks. Both models have shown a similar prediction capacity and they significantly outperformed the results obtained by Abraham *et al*. [7], which is the only work in this area that uses the same compound dataset. To the best of our knowledge this is the largest dataset of VOCs with their associated log P_liver_ values.

A key aspect of the good performance of our approaches is based on the careful selection of the descriptors used to build our models. This selection was first done using an automatic feature selection method, which gives a subset of descriptors where their joint application yields good regression accuracy in a non-linear model. However, many of these descriptors were not easily interpretable. Thereby, a new manual selection of descriptors was done by domain experts aiming at introducing descriptors that model the target property and the differences of the compound families in the dataset. In this way, a smaller and more interpretable subset of descriptors was obtained. While the prediction capacity of this combined subset of descriptors is similar, this smaller subset is preferred as it allows a better understanding of the target property and reduces the likelihood of having a chance correlation due to the small size of the dataset.

This semi-automatic approach can be also applied to model other properties and other compounds, as long as statistical methods and expert knowledge are available. Nevertheless, it is important to always be cautious in the use of QSPR approaches. While prediction accuracy on unseen compounds are estimated by the use of a test set, it is hard to assess the prediction accuracy of the compounds that fall outside of the applicability domain of the model. The applicability domain of a model is usually affected by the training set, the complexity or dimensionality of its representation and the prediction model [32,33]. For these reasons, our model may not perform with the same accuracy for compounds of a different nature to those present in the training set. Yet, the use of strategies that include expert knowledge during the modeling phase leads to more plausible models that are easier to interpret and more likely to better generalize to unseen compounds.

Finally, this work contributes reliable techniques to predict a metric related to exposure to chemicals in the environment, which may be applied to risk assessment and decision making in public health policies.

## Figures and Tables

**Table 1:** Dataset of *in vitro* blood-to-liver partition coefficients for 122 volatile organic compounds [7]. The color coding used in the figures in Section 3.2 is detailed here as follows: alkanes (blue/^b^), alcohols (green/^g^), aromatics (orange/^o^), some halogenated hydrocarbons (red/^r^) and the remaining compounds (white/^w^). Predicted values for decision trees (DT) and neural networks ensemble (NNE) using one sixth of the dataset (Experiment 1, Ntest = 20, Ntrain = 102) and half of the dataset (Experiment 2, Ntest = Ntrain = 61) are reported. Trn or Tst denotes whether the compound was part of the training or test set respectively.

Compound		Log P_liver_	Experiment 1			Experiment 2		
			Set	DT	NNE	Set	DT	NNE
1^w^	Nitrous oxide	−0.04	Trn	−0.101	−0.031	Tst	−0.210	−0.028
2 ^b^	Pentane	0.61	Trn	0.438	0.465	Tst	0.356	0.293
3 ^b^	Hexane	0.48	Trn	0.507	0.567	Trn	0.475	0.460
4 ^b^	Heptane	0.46	Tst	0.568	0.621	Trn	0.577	0.595
5 ^b^	Octane	0.73	Trn	0.684	0.680	Tst	0.687	0.706
6 ^b^	Nonane	0.50	Tst	0.762	0.746	Trn	0.801	0.786
7 ^b^	Decane	0.85	Trn	0.862	0.843	Tst	0.946	0.883
8 ^b^	2-Methylpentane	1.04	Trn	0.789	0.857	Trn	0.702	0.859
9 ^b^	3-Methylpentane	1.06	Tst	0.814	0.898	Trn	0.789	0.916
10 ^b^	3-Methylhexane	0.93	Trn	0.863	0.910	Tst	0.845	0.947
11 ^b^	2-Methylheptane	0.52	Trn	0.713	0.751	Tst	0.724	0.800
12 ^b^	2-Methyloctane	0.74	Trn	0.789	0.807	Tst	0.835	0.864
13 ^b^	2-Methylnonane	0.76	Trn	0.786	0.733	Tst	0.836	0.799
14 ^b^	2,2-Dimethylbutane	1.13	Trn	0.719	0.741	Tst	0.594	0.731
15 ^b^	2,2,4-Trimethylpentane	0.80	Tst	0.579	0.556	Trn	0.528	0.602
16 ^b^	2,3,4-Trimethylpentane	0.70	Trn	0.902	0.886	Tst	1.007	0.976
17 ^w^	Cyclopropane	0.02	Trn	0.118	0.075	Tst	0.082	0.213
18 ^w^	Methylcyclopentane	0.96	Trn	0.888	0.905	Trn	0.906	1.013
19 ^w^	Cyclohexane	0.88	Trn	0.843	0.918	Tst	0.828	0.944
20 ^w^	Methylcyclohexane	0.71	Tst	0.830	0.892	Trn	0.826	0.922
21 ^w^	1,2-Dimethylcyclohexane	1.17	Trn	0.957	0.949	Trn	1.136	1.099
22 ^w^	1,2,4-Trimethylcyclohexane	0.86	Trn	0.886	0.820	Trn	1.020	0.932
23 ^w^	*tert*-Butylcyclohexane	0.30	Trn	0.529	0.447	Trn	0.608	0.407
24 ^w^	JP-10	0.98	Trn	1.083	0.972	Tst	1.452	1.400
25 ^w^	Ethene	0.24	Tst	0.044	0.084	Trn	0.039	0.226
26 ^w^	Propene	−0.07	Trn	0.123	0.092	Tst	0.148	0.228
27 ^w^	1-Octene	0.80	Trn	0.687	0.719	Tst	0.693	0.732
28 ^w^	1-Nonene	0.93	Tst	0.901	0.815	Trn	0.833	0.854
29 ^w^	1-Decene	1.06	Trn	0.981	0.871	Tst	0.952	0.915
30 ^w^	1,3-Butadiene	−0.26	Trn	0.143	0.083	Tst	0.213	0.199
31 ^w^	2-Methyl-1,3-butadiene	0.32	Trn	0.244	0.321	Tst	0.440	0.318
32 ^w^	Difluoromethane	0.24	Trn	−0.068	−0.053	Tst	−0.251	−0.004
33 ^w^	Chloromethane	0.23	Trn	0.013	0.058	Trn	−0.072	0.140
34 ^r^	Dichloromethane	−0.11	Trn	0.059	0.057	Trn	0.002	0.073
35 ^r^	Chloroform	0.13	Trn	0.167	0.141	Tst	0.165	0.099
36 ^w^	Carbon tetrachloride	0.53	Trn	0.520	0.607	Trn	0.472	0.517
37 ^w^	Chloroethane	0.07	Trn	0.085	0.035	Tst	0.049	0.162
38 ^w^	1,1-Dichloroethane	0.15	Trn	0.106	0.031	Tst	0.140	0.133
39 ^w^	1,2-Dichloroethane	0.16	Trn	0.155	0.078	Trn	0.194	0.164
40 ^r^	1,1,1-Trichloroethane	0.44	Trn	0.261	0.238	Trn	0.374	0.287
41 ^r^	1,1,2-Trichloroethane	0.19	Trn	0.230	0.145	Tst	0.231	0.165
42 ^r^	1,1,1,2-Tetrachloroethane	0.40	Trn	0.333	0.288	Tst	0.421	0.252
43 ^r^	1,1,2,2-Tetrachloroethane	0.16	Trn	0.318	0.262	Tst	0.360	0.221
44 ^w^	Pentachloroethane	0.39	Tst	0.406	0.447	Trn	0.435	0.415
45 ^w^	Hexachloroethane	0.81	Trn	0.475	0.714	Trn	0.368	0.835
46 ^w^	1-Chloropropane	0.12	Trn	0.201	0.148	Trn	0.292	0.227
47 ^w^	2-Chloropropane	0.18	Trn	0.163	0.083	Tst	0.171	0.191
48 ^w^	1,2-Dichloropropane	0.25	Trn	0.220	0.112	Trn	0.223	0.165
49 ^w^	Dibromomethane	−0.04	Trn	0.123	0.069	Trn	−0.025	0.039
50 ^r^	1,2-Dibromoethane	0.00	Trn	0.215	0.107	Trn	0.308	0.160
51 ^w^	1-Bromopropane	−0.06	Trn	0.221	0.129	Tst	0.266	0.198
52 ^w^	2-Bromopropane	0.00	Trn	0.201	0.138	Tst	0.293	0.238
53 ^w^	Fluorochloromethane	−0.17	Trn	−0.002	−0.002	Tst	−0.105	0.040
54 ^w^	Bromochloromethane	0.26	Trn	0.092	0.055	Tst	−0.004	−0.007
55 ^w^	Bromodichloromethane	0.00	Trn	0.195	0.144	Tst	0.136	0.085
56 ^w^	Chlorodibromomethane	0.22	Trn	0.224	0.180	Tst	0.104	0.264
57 ^r^	1,1-Dichloro-1-fluoroethane	0.20	Trn	0.214	0.154	Trn	0.257	0.211
58 ^r^	1-Bromo-2-chloroethane	0.03	Trn	0.186	0.095	Trn	0.261	0.150
59 ^r^	2-Chloro-1,1,1-trifluoroethane	0.17	Trn	0.202	0.089	Trn	0.131	0.126
60 ^r^	2;2-Dichloro-1,1,1-trifluoroethane	0.06	Trn	0.288	0.223	Trn	0.246	0.225
61 ^w^	1,1-Difluoroethene	0.64	Trn	0.075	0.051	Trn	0.073	0.155
62 ^w^	Chloroethene	0.03	Trn	0.057	0.058	Tst	0.070	0.162
63 ^r^	1,1-Dichloroethene	−0.05	Tst	0.184	0.189	Trn	0.212	0.207
64 ^r^	*cis*-1,2-Dichloroethene	0.02	Trn	0.064	0.009	Tst	0.057	0.064
65 ^r^	*trans*-1,2-Dichloroethene	0.07	Trn	0.078	0.030	Trn	0.168	0.102
66 ^r^	Trichloroethene	0.27	Trn	0.191	0.133	Trn	0.198	0.123
67 ^w^	Tetrachloroethene	0.66	Trn	0.310	0.320	Tst	0.268	0.264
68 ^r^	Bromoethene	0.03	Tst	0.067	0.029	Trn	0.046	0.056
69 ^r^	1-Chloro-2,2-difluoroethene	−0.02	Tst	0.090	0.001	Trn	0.120	0.070
70 ^w^	1,2-Epoxy-3-butene	−0.23	Trn	−0.018	−0.078	Trn	0.076	−0.008
71 ^g^	1-Propanol	0.05	Trn	−0.020	−0.047	Tst	0.059	0.001
72 ^g^	2-Propanol	−0.03	Trn	−0.048	−0.042	Trn	0.020	−0.007
73 ^g^	1-Butanol	0.02	Tst	0.073	0.115	Trn	0.207	0.114
74 ^g^	2-Methyl-1-propanol	0.02	Trn	0.026	−0.053	Tst	0.011	−0.050
75 ^g^	*tert*-Butanol	0.01	Trn	−0.002	0.100	Trn	0.118	0.098
76 ^g^	1-Pentanol	0.41	Trn	0.398	0.330	Trn	0.285	0.291
77 ^g^	3-Methyl-1-butanol	0.22	Tst	0.408	0.362	Trn	0.320	0.388
78 ^g^	*tert*-Amyl alcohol	0.09	Tst	0.379	0.290	Trn	0.255	0.280
79 ^w^	Acetone	0.02	Trn	−0.148	−0.018	Trn	0.008	−0.029
80 ^w^	Butanone	0.12	Trn	−0.024	0.024	Tst	0.134	0.023
81 ^w^	2-Pentanone	0.13	Trn	0.054	0.093	Trn	0.168	0.081
82 ^w^	4-Methyl-2-pentanone	0.23	Trn	0.426	0.433	Tst	0.368	0.551
83 ^w^	2-Heptanone	0.30	Trn	0.436	0.483	Tst	0.343	0.455
84 ^w^	Methyl acetate	−0.03	Trn	−0.118	−0.166	Tst	−0.089	−0.147
85 ^w^	Ethyl acetate	0.13	Trn	−0.036	−0.002	Tst	0.102	−0.012
86 ^w^	Propyl acetate	0.48	Trn	0.372	0.215	Trn	0.240	0.248
87 ^w^	Isopropyl acetate	0.62	Tst	0.485	0.318	Trn	0.345	0.499
88 ^w^	Butyl acetate	0.51	Trn	0.436	0.478	Trn	0.364	0.524
89 ^w^	Isobutyl acetate	0.73	Trn	0.431	0.470	Trn	0.357	0.540
90 ^w^	Pentyl acetate	0.66	Trn	0.527	0.572	Trn	0.428	0.617
91 ^w^	Isopentyl acetate	0.76	Trn	0.592	0.642	Trn	0.504	0.762
92 ^w^	Diethyl ether	−0.17	Tst	0.043	0.183	Trn	0.251	0.195
93 ^w^	*tert*-Butyl methyl ether	0.17	Trn	0.345	0.201	Tst	0.175	0.161
94 ^w^	*tert*-Butyl ethyl ether	0.45	Trn	0.624	0.634	Tst	0.575	0.869
95 ^w^	*tert*-Amyl methyl ether	0.28	Tst	0.405	0.492	Trn	0.380	0.470
96 ^w^	Divinyl ether	0.07	Trn	−0.072	−0.100	Tst	0.026	−0.059
97 ^w^	Ethylene oxide	−0.07	Trn	−0.146	−0.128	Trn	−0.108	−0.042
98 ^w^	Cyanoethylene oxide	−0.56	Trn	−0.158	−0.205	Tst	−0.168	−0.157
99 ^w^	Halothane	0.29	Trn	0.323	0.290	Trn	0.262	0.298
100 ^w^	Teflurane	0.23	Trn	0.261	0.194	Tst	0.147	0.220
101 ^w^	Fluroxene	0.18	Tst	0.081	−0.042	Trn	0.057	0.019
102 ^w^	Enflurane	0.27	Trn	0.356	0.327	Tst	0.300	0.357
103 ^w^	Isoflurane	0.36	Trn	0.314	0.317	Trn	0.139	0.320
104 ^w^	Sevoflurane	0.63	Trn	0.393	0.354	Tst	0.281	0.413
105 ^w^	Methoxyflurane	0.19	Trn	0.247	0.234	Trn	0.105	0.241
106 ^w^	1-nitropropane	−0.13	Trn	0.084	−0.052	Tst	0.056	−0.005
107 ^w^	2-nitropropane	−0.43	Trn	0.056	−0.088	Trn	0.015	−0.033
108 ^w^	Carbon disulfide	0.48	Trn	0.151	0.298	Tst	0.012	0.142
109 ^o^	Benzene	0.21	Trn	0.182	0.108	Tst	−0.005	0.121
110 ^o^	Toluene	0.50	Trn	0.279	0.275	Tst	0.137	0.194
111 ^o^	Ethylbenzene	0.31	Tst	0.310	0.329	Trn	0.157	0.276
112 ^o^	*o*-Xylene	0.34	Trn	0.399	0.364	Tst	0.428	0.281
113 ^o^	*m*-Xylene	0.37	Trn	0.306	0.334	Tst	0.144	0.281
114 ^o^	*p*-Xylene	0.34	Trn	0.406	0.367	Trn	0.391	0.285
115 ^o^	1,2,4-Trimethylbenzene	0.43	Trn	0.414	0.370	Tst	0.481	0.288
116 ^o^	*tert*-Butylbenzene	0.49	Trn	0.434	0.368	Tst	0.492	0.276
117 ^o^	Styrene	0.47	Trn	0.314	0.298	Tst	0.329	0.246
118 ^o^	*m*-Methylstyrene	0.23	Trn	0.364	0.326	Trn	0.341	0.272
119 ^o^	*p*-Methylstyrene	0.14	Tst	0.441	0.409	Trn	0.533	0.298
120 ^w^	Chlorobenzene	0.31	Trn	0.318	0.316	Tst	0.232	0.225
121 ^w^	4-Chlorobenzotrifluoride	0.28	Trn	0.519	0.418	Tst	0.572	0.443
122 ^w^	Furan	−0.05	Trn	0.033	−0.053	Tst	−0.038	0.067

**Table 2:** Correlation coefficient of Se *vs.* AMW, Pol, ALOGP and Mor29u.

Descriptor	*r* (correlation coefficient of Se *vs*. descriptor)	
	Se ≤ 16.025	Se > 16.025
AMW	−0.55	−0.33
Pol	0.57	0.32
ALOGP	0.12	0.75
Mor29u	0.09	−0.15

**Table 3:** Final set of selected descriptors.

Descriptor	Meaning	Family
AMW	average molecular weight	Constitutional
Mor29u	3D-MoRSE - signal 29/unweighted	3D-MoRSE
ALOGP	Ghose-Crippen octanol-water partition coeff. (logP)	Molecular properties
Pol	polarity number	Topological
Se	sum of atomic Sanderson electronegativities	Constitutional

## Supplementary Materials

Supplementary materials can be accessed at: http://www.mdpi.com/1420-3049/17/12/14937/s1.
