# Supplementary material for: QSPR Models for Predicting Log Pliver Values for Volatile Organic Compounds Combining Statistical Methods and Domain Knowledge
Source: Molecules. 2012 Dec 17;17(12):14937–53. doi: 10.3390/molecules171214937 (PMC6268846; doi:10.3390/molecules171214937)
Supplement: Supplementary file 1 [file molecules-17-14937-s001.pdf]

Supplementary Material

Table S1. Prediction results for decision trees and neural network ensemble using 16.6% of the compounds for testing.

Compound		Log P_{liver}	Decision Tree	Neural Network Ensemble
4	Heptane	0.46	0.568	0.621
6	Nonane	0.50	0.762	0.746
9	3-Methylpentane	1.06	0.814	0.898
15	2,2,4-Trimethylpentane	0.80	0.579	0.556
20	Methylcyclohexane	0.71	0.83	0.892
25	Ethene	0.24	0.044	0.084
28	1-Nonene	0.93	0.901	0.815
44	Pentachloroethane	0.39	0.406	0.447
63	1,1-Dichloroethene	−0.05	0.184	0.189
68	Bromoethene	0.03	0.067	0.029
69	1-Chloro-2,2-difluoroethene	−0.02	0.09	0.001
73	1-Butanol	0.02	0.073	0.115
77	3-Methyl-1-butanol	0.22	0.408	0.362
78	*tert*-Amyl alcohol	0.09	0.379	0.29
87	Isopropyl acetate	0.62	0.485	0.318
92	Diethyl ether	−0.17	0.043	0.183
95	*tert*-Amyl methyl ether	0.28	0.405	0.492
101	Fluroxene	0.18	0.081	−0.042
111	Ethylbenzene	0.31	0.31	0.329
119	p-Methylstyrene	0.14	0.441	0.409

Table S2. Prediction results for decision trees and neural network ensemble using 50% of the compounds for testing.

Compound		Log P_{liver}	Decision Tree	Neural Network Ensemble
1	Nitrous oxide	−0.04	−0.210	−0.028
2	Pentane	0.61	0.356	0.293
5	Octane	0.73	0.687	0.706
7	Decane	0.85	0.946	0.883
10	3-Methylhexane	0.93	0.845	0.947
11	2-Methylheptane	0.52	0.724	0.800
12	2-Methyloctane	0.74	0.835	0.864
13	2-Methylnonane	0.76	0.836	0.799
14	2,2-Dimethylbutane	1.13	0.594	0.731
16	2,3,4-Trimethylpentane	0.70	1.007	0.976
17	Cyclopropane	0.02	0.082	0.064
19	Cyclohexane	0.88	0.828	0.944
24	JP-10	0.98	1.452	1.400
26	Propene	−0.07	0.148	0.228
27	1-Octene	0.80	0.693	0.732
29	1-Decene	1.06	0.952	0.915
30	1,3-Butadiene	−0.26	0.213	0.199

Table S2. *Cont.*

Compound		Log P_{liver}	Decision Tree	Neural Network Ensemble
31	2-Methyl-1,3-butadiene	0.32	0.440	0.318
32	Difluoromethane	0.24	−0.251	−0.004
35	Chloroform	0.13	0.165	−0.012
37	Chloroethane	0.07	0.049	0.162
38	1,1-Dichloroethane	0.15	0.140	0.133
41	1,1,2-Trichloroethane	0.19	0.231	0.165
42	1,1,1,2-Tetrachloroethane	0.40	0.421	0.252
43	1,1,2,2-Tetrachloroethane	0.16	0.36	0.221
47	2-Chloropropane	0.18	0.171	0.191
51	1-Bromopropane	−0.06	0.266	0.198
52	2-Bromopropane	0.00	0.293	0.085
53	Fluorochloromethane	−0.17	−0.105	0.040
54	Bromochloromethane	0.26	−0.004	−0.007
55	Bromodichloromethane	0.00	0.136	0.238
56	Chlorodibromomethane	0.22	0.104	0.264
62	Chloroethene	0.03	0.070	0.162
64	*cis*-1,2-Dichloroethene	0.02	0.057	−0.050
67	Tetrachloroethene	0.66	0.268	0.264
71	1-Propanol	0.05	0.059	0.001
74	2-Methyl-1-propanol	0.02	0.011	0.213
80	Butanone	0.12	0.134	0.023
82	4-Methyl-2-pentanone	0.23	0.368	0.220
83	2-Heptanone	0.30	0.343	0.455
84	Methyl acetate	−0.03	−0.089	−0.147
85	Ethyl acetate	0.13	0.102	0.099
93	*tert*-Butyl methyl ether	0.17	0.175	0.161
94	*tert*-Butyl ethyl ether	0.45	0.575	0.869
96	Divinyl ether	0.07	0.026	−0.059
98	Cyanoethylene oxide	−0.56	−0.168	−0.157
100	Teflurane	0.23	0.147	0.551
102	Enflurane	0.27	0.300	0.357
104	Sevoflurane	0.63	0.281	0.413
106	1-nitropropane	−0.13	0.056	−0.005
108	Carbon disulfide	0.48	0.012	0.142
109	Benzene	0.21	−0.005	0.121
110	Toluene	0.50	0.137	0.194
112	*o*-Xylene	0.34	0.428	0.281
113	*m*-Xylene	0.37	0.144	0.281
115	1,2,4-Trimethylbenzene	0.43	0.481	0.288
116	*tert*-Butylbenzene	0.49	0.492	0.276
117	Styrene	0.47	0.329	0.246
120	Chlorobenzene	0.31	0.232	0.225
121	4-Chlorobenzotrifluoride	0.28	0.572	0.443
122	Furan	−0.05	−0.038	0.067

Table S3. Values of model descriptors (Se, Pol, ALOGP, AMW, Mor29u) and target (log P_{liver}) for all molecules sorted by ascending Se value.

Compound		Log P_{liver}	Se	Pol	ALOGP	AMW	Mor29u
108	Carbon disulfide	0.48	3.15	0	1.651	25.38	−0.001
1	Nitrous oxide	−0.04	3.65	0	0.247	14.67	0.051
33	Chloromethane	0.23	5.09	0	0.846	10.1	0.017
49	Dibromomethane	−0.04	5.23	0	1.496	34.77	0.032
54	Bromochloromethane	0.26	5.32	0	1.284	25.88	0.005
34	Dichloromethane	−0.11	5.41	0	1.073	16.99	−0.012
56	Chlorodibromomethane	0.22	5.55	0	2.025	41.65	−0.012
53	Fluorochloromethane	−0.17	5.6	0	0.775	13.7	0.038
55	Bromodichloromethane	0.00	5.64	0	1.821	32.76	−0.045
35	Chloroform	0.13	5.74	0	1.617	23.87	−0.076
25	Ethene	0.24	5.77	0	0.947	4.68	−0.04
32	Difluoromethane	0.24	5.79	0	0.477	10.41	0.113
68	Bromoethene	0.03	6	0	1.086	17.83	−0.033
36	Carbon tetrachloride	0.53	6.06	0	3.585	30.76	−0.137
62	Chloroethene	0.03	6.09	0	1.002	10.42	−0.053
63	1,1-Dichloroethene	−0.05	6.41	0	1.688	16.16	−0.095
64	*cis*-1,2-Dichloroethene	0.02	6.41	1	1.057	16.16	−0.038
65	*trans*-1,2-Dichloroethene	0.07	6.41	1	1.057	16.16	−0.109
66	Trichloroethene	0.27	6.74	2	1.743	21.9	−0.079
61	1,1-Difluoroethene	0.64	6.79	0	1.114	10.67	−0.041
67	Tetrachloroethene	0.66	7.06	4	2.429	27.64	−0.075
97	Ethylene oxide	−0.07	7.09	0	−0.131	6.29	−0.006
69	1-Chloro-2,2-difluoroethene	−0.02	7.12	2	1.169	16.41	−0.064
37	Chloroethane	0.07	7.97	0	1.194	8.07	−0.009
50	1,2-Dibromoethane	0.00	8.11	1	1.801	23.48	−0.132
58	1-Bromo-2-chloroethane	0.03	8.2	1	1.655	17.93	−0.111
38	1,1-Dichloroethane	0.15	8.3	0	1.251	12.37	−0.06
39	1,2-Dichloroethane	0.16	8.3	1	1.51	12.37	−0.077
98	Cyanoethylene oxide	−0.56	8.31	2	−0.158	8.63	0.042
40	1,1,1-Trichloroethane	0.44	8.62	0	2.029	16.67	−0.154
41	1,1,2-Trichloroethane	0.19	8.62	2	1.955	16.67	−0.068
17	Cyclopropane	0.02	8.65	0	1.369	4.68	−0.012
26	Propene	−0.07	8.65	0	1.35	4.68	−0.055
57	1,1-Dichloro-1-fluoroethane	0.20	8.81	0	1.833	14.62	−0.091
42	1,1,1,2-Tetrachloroethane	0.40	8.95	3	2.443	20.98	−0.153
43	1,1,2,2-Tetrachloroethane	0.16	8.95	4	2.399	20.98	−0.117
122	Furan	−0.05	9.09	0	0.934	7.56	0.039
44	Pentachloroethane	0.39	9.27	6	2.888	25.28	−0.129
59	2-Chloro-1,1,1-trifluoroethane	0.17	9.51	3	1.853	14.81	−0.003
45	Hexachloroethane	0.81	9.59	9	3.376	29.59	−0.051
30	1,3-Butadiene	−0.26	9.65	1	1.415	5.41	−0.083
99	Halothane	0.29	9.74	6	2.509	24.67	−0.04
60	2,2-Dichloro-1,1,1-trifluoroethane	0.06	9.84	6	2.298	19.12	−0.043

Table S3. *Cont.*

Compound		Log P_{liver}	Se	Pol	ALOGP	AMW	Mor29u
100	Teflurane	0.23	9.93	6	2.211	22.62	0.015
79	Acetone	0.02	9.98	0	−0.244	5.81	−0.06
51	1-Bromopropane	−0.06	10.76	1	1.864	11.18	−0.076
52	2-Bromopropane	0.00	10.76	0	1.717	11.18	−0.103
46	1-Chloropropane	0.12	10.86	1	1.718	7.14	−0.101
47	2-Chloropropane	0.18	10.86	0	1.572	7.14	−0.034
70	1,2-Epoxy-3-butene	−0.23	10.98	2	0.515	6.37	−0.043
96	Divinyl ether	0.07	10.98	2	0.219	6.37	−0.031
48	1,2-Dichloropropane	0.25	11.18	2	1.887	10.27	−0.043
84	Methyl acetate	−0.03	11.31	2	0.021	6.74	0.032
109	Benzene	0.21	11.65	3	1.83	6.51	0.102
71	1-Propanol	0.05	11.86	1	0.515	5.01	−0.024
72	2-Propanol	−0.03	11.86	0	0.368	5.01	−0.009
120	Chlorobenzene	0.31	11.97	5	2.494	9.38	−0.001
31	2-Methyl-1,3-butadiene	0.32	12.53	2	1.861	5.24	−0.17
80	Butanone	0.12	12.86	2	0.423	5.55	−0.068
106	1-nitropropane	−0.13	13.41	3	1.164	6.85	0.036
107	2-nitropropane	−0.43	13.41	4	1.018	6.85	0.052
105	Methoxyflurane	0.19	13.53	9	2.143	13.75	0.071
85	Ethyl acetate	0.13	14.19	3	0.37	6.29	−0.039
101	Fluroxene	0.18	14.4	5	1.142	9.7	0.043
110	Toluene	0.50	14.53	5	2.316	6.14	0.072
73	1-Butanol	0.02	14.75	2	0.971	4.94	−0.06
74	2-Methyl-1-propanol	0.02	14.75	2	0.834	4.94	0.055
75	tert-Butanol	0.01	14.75	0	0.573	4.94	−0.03
92	Diethyl ether	−0.17	14.75	2	0.748	4.94	−0.103
102	Enflurane	0.27	14.75	11	2.667	15.37	−0.006
103	Isoflurane	0.36	14.75	10	2.534	15.37	0.087
117	Styrene	0.47	15.53	7	2.382	6.51	−0.036
81	2-Pentanone	0.13	15.75	3	0.879	5.38	−0.032
2	Pentane	0.61	16.3	2	2.653	4.25	−0.028
121	4-Chlorobenzotrifluoride	0.28	16.4	13	3.437	12.04	−0.112
86	Propyl acetate	0.48	17.07	4	0.894	6.01	−0.064
87	Isopropyl acetate	0.62	17.07	4	0.747	6.01	−0.141
18	Methylcyclopentane	0.96	17.3	2	2.533	4.68	−0.376
19	Cyclohexane	0.88	17.3	3	2.737	4.68	−0.313
111	Ethylbenzene	0.31	17.42	7	2.772	5.9	0.117
112	*o*-Xylene	0.34	17.42	8	2.802	5.9	−0.053
113	*m*-Xylene	0.37	17.42	7	2.802	5.9	0.127
114	*p*-Xylene	0.34	17.42	7	2.802	5.9	−0.03
76	1-Pentanol	0.41	17.63	3	1.427	4.9	−0.052
77	3-Methyl-1-butanol	0.22	17.63	3	1.223	4.9	−0.088
78	*tert*-Amyl alcohol	0.09	17.63	3	1.097	4.9	−0.055
93	*tert*-Butyl methyl ether	0.17	17.63	3	0.982	4.9	−0.012
104	Sevoflurane	0.63	18.33	15	2.233	13.34	0.011

Table S3. *Cont.*

Compound		Log P_{liver}	Se	Pol	ALOGP	AMW	Mor29u
118	*m*-Methylstyrene	0.23	18.42	9	2.868	6.22	0.016
119	*p*-Methylstyrene	0.14	18.42	9	2.868	6.22	−0.106
82	4-Methyl-2-pentanone	0.23	18.63	4	1.131	5.27	−0.115
3	Hexane	0.48	19.19	3	3.109	4.31	−0.046
8	2-Methylpentane	1.04	19.19	3	2.904	4.31	−0.204
9	3-Methylpentane	1.06	19.19	4	2.904	4.31	−0.259
14	2,2-Dimethylbutane	1.13	19.19	3	2.654	4.31	−0.152
88	Butyl acetate	0.51	19.96	5	1.35	5.81	−0.085
89	Isobutyl acetate	0.73	19.96	5	1.213	5.81	−0.09
20	Methylcyclohexane	0.71	20.19	5	2.989	4.68	−0.267
115	1,2,4-Trimethylbenzene	0.43	20.3	10	3.289	5.72	−0.027
94	*tert*-Butyl ethyl ether	0.45	20.51	4	1.331	4.87	−0.215
95	*tert*-Amyl methyl ether	0.28	20.51	6	1.506	4.87	−0.08
83	2-Heptanone	0.30	21.51	5	1.792	5.19	−0.028
4	Heptane	0.46	22.07	4	3.565	4.36	−0.053
10	3-Methylhexane	0.93	22.07	5	3.361	4.36	−0.237
90	Pentyl acetate	0.66	22.84	6	1.806	5.66	−0.068
91	Isopentyl acetate	0.76	22.84	6	1.602	5.66	−0.13
21	1,2-Dimethylcyclohexane	1.17	23.07	8	3.241	4.68	−0.42
27	1-Octene	0.80	23.07	5	3.631	4.68	−0.113
116	*tert*-Butylbenzene	0.49	23.19	11	3.23	5.59	−0.011
5	Octane	0.73	24.95	5	4.021	4.39	−0.065
11	2-Methylheptane	0.52	24.95	5	3.817	4.39	−0.102
15	2,2,4-Trimethylpentane	0.80	24.95	5	3.362	4.39	−0.008
16	2,3,4-Trimethylpentane	0.70	24.95	8	3.408	4.39	−0.309
24	JP-10	0.98	25.07	10	3.001	5.24	−0.618
22	1,2,4-Trimethylcyclohexane	0.86	25.95	10	3.493	4.68	−0.302
28	1-Nonene	0.93	25.95	6	4.087	4.68	−0.144
6	Nonane	0.50	27.84	6	4.477	4.42	−0.08
12	2-Methyloctane	0.74	27.84	6	4.273	4.42	−0.115
23	*tert*-Butylcyclohexane	0.30	28.84	11	3.903	4.68	0.014
29	1-Decene	1.06	28.84	7	4.543	4.68	−0.162
7	Decane	0.85	30.72	7	4.934	4.45	−0.114
13	2-Methylnonane	0.76	30.72	7	4.729	4.45	−0.058
